# Perception of geometric sequences and numerosity both predict formal geometric competence in primary school children

**DOI:** 10.1038/s41598-021-93710-x

**Published:** 2021-07-09

**Authors:** Elisa Castaldi, Roberto Arrighi, Guido M. Cicchini, Arianna Andolfi, Giuseppe Maduli, David C. Burr, Giovanni Anobile

**Affiliations:** 1 Department of Translational Research and New Technologies in Medicine and Surgery, University of Pisa, 56126 Pisa, Italy; 2 Department of Neuroscience, Psychology, Pharmacology, and Child Health, University of Florence, 50139 Florence, Italy; 3 CNR Neuroscience Institute, 56100 Pisa, Italy

**Keywords:** Psychology, Human behaviour

## Abstract

While most animals have a sense of number, only humans have developed symbolic systems to describe and organize mathematical knowledge. Some studies suggest that human arithmetical knowledge may be rooted in an ancient mechanism dedicated to perceiving numerosity, but it is not known if formal geometry also relies on basic, non-symbolic mechanisms. Here we show that primary-school children who spontaneously detect and predict geometrical sequences (non-symbolic geometry) perform better in school-based geometry tests indexing formal geometric knowledge. Interestingly, numerosity discrimination thresholds also predicted and explained a specific portion of variance of formal geometrical scores. The relation between these two non-symbolic systems and formal geometry was not explained by age or verbal reasoning skills. Overall, the results are in line with the hypothesis that some human-specific, symbolic systems are rooted in non-symbolic mechanisms.

## Introduction

Humans are the only animals to develop symbolic systems to express knowledge, one of which is geometry. The word *geometry* etymologically means “measurement of earth” and is an important branch of mathematics, together with arithmetic. Geometric and arithmetic knowledge are an essential part of school curricula and widely used in everyday life, necessary to guide navigation and to decipher the abundant numerical information characterizing the environment. Despite their important role in our everyday life, the origin of formal geometrical and arithmetical knowledge is still largely debated.

One influential theory holds that symbolic arithmetical abilities may be rooted in an ancient non-symbolic system devoted to perceiving numerical quantities^1–3^. This system is not human-specific^4^, seems to be functional from the first hours of life^5–7^, even in premature newborns^8^, and present in indigenous populations with scarce school experience and restricted language for numbers^9^. Although subject to criticism^10,11^, this theory has received support from a large body of research showing that children with higher arithmetical abilities are also more precise in evaluating relative numerosity^2,12,13^, while children with a specific deficit in math acuity (dyscalculia) are poor on numerosity tasks^14–17^. However, alternative accounts have also been offered for the relationship between non-symbolic and symbolic numerical abilities^18,19^ (see ref^17^ for a recent review of a more comprehensive view of dyscalculia).

**Figure 1:**
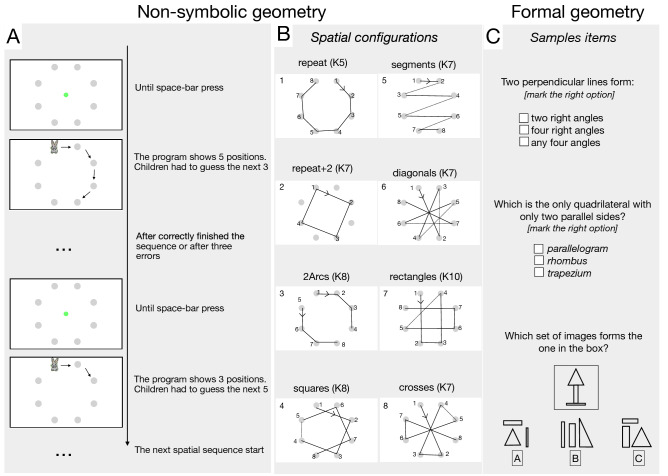
Non-symbolic and formal geometry tasks. (**A**) Schematic representation of the procedure to assess non-symbolic geometry (the “repeat” spatial sequence is depicted). (**B**) Representation of the eight spatial configurations tested. The numbers near the circles represent the order followed by the flashing stimulus. The numbers in the boxes represent the presentation order. Complexity (K) is indicated for each sequence. (**C**) Three examples of items measuring formal geometrical abilities (reproduced with permission).

In a similar vein, researchers have investigated the existence of a homologous non-symbolic and preverbal geometrical intuition that would pave the way for the development of formal geometry. Several studies have shown that a few days from birth, human infants are sensitive to many geometrical cues including angular size^20^, shape^21,22^, relative length^23^ (likely to underlie changes in the detection of angles and shapes) and object distance from landmarks^24^. Some primitive geometrical intuitions are present also in Amazonian indigenous of the *Munduruku* population, although these people do not have formal instruction in geometry, and a very limited language for geometrical properties^25^. Indigenous adults and children were able to indicate the “odd-one” out of six images, the one lacking a particular geometric property such as parallelism, with almost the same proficiency as age-matched, math-educated Americans, suggesting that they were able to spontaneously detect shape similarities based on some geometrical properties. More recently, the same group of researchers proposed a new paradigm to measure non-symbolic geometrical sensitivity. Amalric et al.^26^ devised a task in which a dot sequentially flashed across eight possible positions around an octagon (see Fig. 1). Participants were first shown a sequence of five locations and then asked to predict the future locations. The sequences followed different geometrical paths that varied in complexity, from simple geometrical primitives (circles and squares) to more sophisticated shapes. Most of the geometrical structures were quickly perceived and exploited to solve the task, suggesting the spontaneous use of a non-symbolic language of geometry. Importantly, not only educated adults, but also pre-schoolers and indigenous people (Munduruku), with poor formal education and restricted language for geometry and numbers, were able to perform this task. Overall, these results point to the existence of an ancient and probably spontaneous, language- and education-independent intuition of geometry.

One interesting question is whether non-symbolic geometrical abilities are related to symbolic geometry. Previous studies have investigated how children use non-symbolic geometrical cues during symbolic map-based navigation^27–29^. For example, the performance of four-year-old pre-schoolers on a non-symbolic odd-one-out task similar to the one described above^25^ as well as performance in a navigation task in which children had to reorient in a 3D environment, were positively correlated with the ability to use symbolic maps to locate targets^28^. Performance in the two non-symbolic tasks did not correlate with each other, suggesting two distinct systems, one for navigation and one for object shape analysis, relying on different geometric properties. These two non-symbolic systems may act as modules of a larger network that includes magnitude perception^30^. Indeed, Ayzenberg and Lourenco^30^ found that magnitude perception, quantified with an area discrimination task, correlated both with the navigation task and the odd-one-out shape identification task, although performance in the navigation and shape identification tasks did not correlate with each other. The authors suggested that navigation ability and form analysis may be indirectly linked through the magnitude perception system (subserving the ability for area discrimination).

Compared with the studies investigating symbolic map-based navigation, only a few have tested how non-symbolic geometric abilities relate to the kind of symbolic formal geometry usually learnt at school. Giofrè et al.^31^ showed that performance on the odd-one-out shape identification task in eighteen-year-olds correlated positively with scores in a paper-and-pencil test of advanced geometrical problems. Lourenco et al.^32^ went further by testing whether a single, aspecific system subserves both formal arithmetical and geometrical learning, or whether these different domains are supported by multiple systems, by measuring the relative contribution of sensitivity to numerosity and cumulative area discrimination to explaining variance in formal arithmetic and geometry performance in adults. Cumulative area sensitivity was taken as an index of non-symbolic geometry, while symbolic geometry was indexed by paper and pencil tasks assessing understanding of geometrical concepts and operations. The results revealed that numerosity sensitivity explained a unique portion of the variance in arithmetical performance, while cumulative area sensitivity specifically contributed to geometrical skills (over and above numerical sensitivity). Interestingly, in 5–6 year-old children sensitivity to both numerosity and area perception explained calculation and geometry variance^33^.

Overall, these results suggest that the development of formal symbolic geometry relies on non-symbolic geometry, as much as a non-symbolic system for numerosity perception seems to act as a start-up tool for formal arithmetic learning^3^. They also suggest that the degree of independence between non-symbolic systems and the specificity of their predictive links with the respective symbolic competences vary depending on the developmental stage.

The link between non-symbolic and symbolic formal geometry achievement has been suggested also by a study^34^ that potentiated emerging skills in geometry and number through games. For 4 months, children played with a math game which included both comparison and manipulation of numerical magnitudes, as well as identification of a deviant shape based on geometric properties or to place objects at locations indicated by a map. Non-symbolic and formal abilities were then evaluated: non-symbolic abilities were quantified by measuring performance in approximate numerical comparison or deviant shape identification tasks, while evaluation of formal competence included testing knowledge of number words and shape names, ability to manipulate numbers presented as words or symbols and ability to answer verbal questions concerning shape similarity and symmetry. The results showed that, compared with the no-training or control social training groups, children trained with the math game showed a marked improvement in both non-symbolic and formal numerical and geometric skills, although the effect on formal skills was less enduring than that on non-symbolic skills, and turned to be not significant when evaluated after 6-12 months.

Unlike the studies discussed above, the paradigm designed by Amalric et al.^26^ requires participants to dynamically extrapolate the next location in sequence by spontaneously identifying a geometrical shape or rule and applying it to reproduce a memorized complex temporal sequence. This paradigm has a child-friendly game-like interface, making it a particularly useful tool to assess knowledge of non-symbolic geometrical in children. However, it is not known whether this measure of informal geometrical intuition is predictive of formal geometry. The aim of the current study was two-fold: to investigate the link between intuitive, non-symbolic geometry, as measured by Amalric et al.’s test, and proficiency at formal (symbolic) geometry (both verbal knowledge and visuospatial abilities) in primary school children; and to test whether formal geometrical skills are also predicted by numerosity, shown by some studies to predict arithmetical skills. We further investigated whether performance in non-symbolic geometry and numerosity tasks independently predict formal geometrical skills, when other possible confounds (such as age and verbal reasoning) are controlled.

## Material and methods

### Participants

For this study we recruited 110 participants (3rd, 4th and 5th grades, 7–11 years old), from two local primary schools, and collected informed consent forms signed by their parents. The testing started in one school at the end of February 2020. Unfortunately, after a few weeks, due to fast escalation of the Covid-19 pandemic, all Italian schools were closed. At that point we had administered the full task protocols to a sample of ~ 50 subjects. Given the complex Italian situation and the information provided by the government, the possibility to continue school testing sessions within a reasonable time window was extremely unlikely. For this reason, the final sample included here is N49 (see the dedicated participant section).

We ran an a priori power analyses for zero-order correlations, estimating effect size by averaging zero-order correlation coefficients extracted from three similar previous studies^31,32,35^. With an effect size of 0.34, a required power of 0.75 and a one-tailed hypothesis (as all the study found a directional positive correlation), the total required sample size was 42.

A total of 49 children (8–10.9 years old, mean = 9.5 years; 28 females, 21 males; 20 third graders, 11 fourth graders, 18 fifth graders) participated in this study (see Table 1). Only those who returned a signed consent from parents were included. Experimental procedures were approved by the local ethics committee (*Comitato Etico Pediatrico Regionale, Azienda Ospedaliero–Uni versitaria Meyer*, Florence, Italy), and are in line with the declaration of Helsinki. Some children did not complete all the tasks because they were unavailable in one of the two testing days (missing data were left empty and excluded by pairwise procedure). The sample size for each test is reported in Table 1.

**Table 1:** Descriptive statistics of the sample characteristics and results.

	Age	Verbal reasoning	Numerosity	Non-symbolic geometry		Formal geometry		
		similarities WISC IV (weighted score M 10, SD 1.5)	Normalized sensitivity	1st run (accuracy)	2nd run (accuracy)	Knowledge factor (Z-score)	Visuo-spatial factor (Z-score)	Combined index (Z-score)
Mean	9.5	12.9	4.19	0.68	0.74	− 0.20	− 0.05	− 0.13
Standard deviation	0.91	3.11	1.39	0.147	0.13	1.17	1.06	0.977
N	49	49	47	49	49	42	42	42

The table shows children age and scores on the different tests evaluating verbal reasoning, numerosity discrimination, non-symbolic and formal geometry. Sample size is also specified.

### General procedures

Stimuli for psychophysical tests were generated with MATLAB 8.1 using PsychToolbox routines. The stimuli were presented on 12.3″ touchscreen tablets (Microsoft Surface Pro), with 2736 × 1824 resolution at a refresh rate of 60 Hz. Each participant was tested in two separate sessions (usually within the same week) lasting about 1 h each. In the first session, formal geometrical abilities were measured by an Italian standardized paper-and-pencil test. The battery was administered collectively by dividing the sample into groups of about twenty students each, organized into four children for each desk. Five adults, (two teachers and three experimenters) were present during the administration in order to avoid communication between children and to provide information when appropriate. Although response times were not measured, the maximum duration of administration was about 50 min. In the second session the same experimenters, naïve of the child’s performance on the test for formal geometrical abilities, administered the psychophysical (non-symbolic) tests singularly to every child, in a pseudorandom order between participants. In addition, a measure of verbal reasoning via the similarities subtest of the WISC-IV battery was achieved as the very last test.

### Data analysis

Data were analysed with both parametric tests (t-tests, zero order and partial correlations, two-tailed) as well as by non-parametric Bootstrap techniques. Data were analysed with Jasp (version 0.8.6 The JASP Team 2020, https://jasp-stats.org), Matlab R2017B (https://it.mathworks.com) and SPSS v.25 (https://www.ibm.com) software. Graphs were created with OriginPro 2015 (https://www.originlab.com/).

### Cognitive geometry battery

We used a paper-and-pencil Italian standardized battery for the assessment of geometry learning in children (see Fig. 1C for sample items)^36^. The battery comprises 8 items (questions) measuring the factor “Geometry knowledge” and 24 items (tables) measuring the factor “Visuo-spatial abilities”. For each item children had to choose the only correct option out of three possibilities. The only exception is for the question “What are these figures called?” in which 3rd graders name six figures. We counted separately for each factor the number of correct responses and transformed them into z-scores (according to the age-appropriate norms provided by the test itself: see Table 1 for descriptive statistics).

The “geometry knowledge” factor collapses questions designed to measure geometrical lexical knowledge and knowledge about figure properties. The questions for 3rd graders were: (1) What are these figures called? (triangle, rhombus, trapezoid, parallelepiped, cylinder, pyramid). (2) What is this line called? (segment). (3) Which of the following drawings contain the figure of a circle? (4) Which of the following figures is not a rectangle? (5) Properties of perpendicular lines. (6) Which of the following figures is not a triangle? (7) Which pair of figures is symmetrical? (8) Which of these is the solid formed by polygons?

For 4th and 5th graders the questions were: (1) What is a segment? (2) What is a protractor? (3) What kind of triangle is represented in the figure? (4) Which of these three figures is concave? (5) With which of these measurements can you construct a scalene triangle? (6) What is the only quadrilateral with only two parallel sides? (7) What kind of triangle is represented in the figure? (8) Characteristics of a parallelogram.

The “visuo-spatial abilities” factor includes exercises designed to measure children’s ability to manipulate geometrical figures. For all the classes, the exercises were the same but with simplified figures for 3rd graders. The visuo-spatial exercises were: Imagine playing with blocks that form a figure. Observe the figure formed with the blocks inside the box and find the one composed by the same blocks (4 items). In this exercise you will see two figures inside a box. Your task is to try to join them together so that you can find out which figure they form (4 items). Imagine a sheet of paper with the same shape as the figure in the box and imagine folding the sheet at the dotted lines. Once folded, imagine joining it together in all its parts. Which of the three alternatives represents the composition of the figure? (4 items). Count the number of cubes that form this figure (4 items). Look for a simple image in the more complex one. For example, look for the triangle you see on the left in the figure on the right (4 items). Look for the surface where all the figures intersect and colour it (4 items).

The reliability levels (Cronbach’s alpha) reported by the test manual were: for the “Geometry knowledge” factor 0.50 for the 3rd graders and 0.46 for 4rt and 5th graders. For the “visuo-spatial abilities” factor Cronbach’s alpha levels were 0.74 for the 3rd graders and 0.72 for 4rt and 5th graders. The reliability levels (Cronbach’s alpha) calculated from the current data for the “Geometry knowledge” factor were 0.67 for the 3rd graders and 0.75 for 4rt and 5th graders. For the “Visuo-spatial abilities”, factor Cronbach’s alpha levels were 0.78 for the 3rd graders and 0.73 for 4th and 5th graders. Despite the small discrepancies between the Cronbach’s values calculated in the symbolic geometry assessments and those reported by the manual (likely due to differences in the sample size), all tests provided good reliability levels.

### Psychophysical non-symbolic geometry test

Non-symbolic, intuitive geometry was measured with an adapted version of the paradigm devised by Amalric, et al.^26^ to test pre-schoolers. Each trial started with a screen showing a symmetrical circular grid (9° diameter) of eight grey dots, which remained visible for the entire experiment. Each trial tested a specific sequence of spatial locations, comprising two separate blocks (1st and 2nd run hereafter). In the 1st run a figure of an animal (different each trial, approx. size 1.5°), flashed on five successive locations. Each flash lasted (1000 ms) with a fixed inter-stimulus interval of (300 ms). After these five jumps the animal disappeared. Children were asked to show the animal’s position in the next three locations, following the geometrical features of the path they had previously seen. Each wrong response was followed by a short sound, then the program automatically restarted from the beginning of the sequence including the right position where the mistaken had been. Children were then asked to point to the next locations to complete the whole sequence. Once finished the 1st run, the screen turned blank and after the experimenter pressed the space bar of an external keyboard, the 2nd run started. This was the same as the 1st but only the first three locations were cued by the program. Once this 2nd run finished, the successive trial started, testing a different spatial sequence (Fig. 1A).

In the entire experiment eight different spatial configurations were tested (Fig. 1B, see main text). Each configuration has an associated degree of complexity (K) that reflects an index of geometrical regularity, with higher values indicating less regularity, thus higher complexity (for full description of the computational procedures to calculate K see refernce^26^). Unlike the original study, in which the different sequences were presented in random order, the eight spatial configurations tested in the current study were presented in the same blocked order to each child, according to the increased difficulty as measured by 5-year-old children in Amalric et al.’s study^26^. The order of the sequences was: repeat (K5), repeat + 2 (K7), 2arcs (K8), squares (K8), segments (K7), diagonals (K7), rectangles (K10), crosses (K7).

Given the correlational nature of the current study, and that most of the spatial configurations have a different degree of complexity, the blocked order procedure was chosen to reduce possible difficulties in controlling for inter-subject variability in performance induced by hysteresis (the influence of the difficulty of the previous N-1 session on the following one). As in Amalric et al.’s^26^ study, to reduce the duration of the experiment, children were tested with a single exemplar of each sequence specifically with the animal moving clockwise, as shown by the arrows in Fig. 1A. While in Amalric et al.’s^26^ study the “4-segments” sequence was presented 4 times, in order to test all 4 axial symmetries, in the current study it was presented only once.

In the non-symbolic geometry test, subject performance was calculated as proportion of correct responses. Performance was independently calculated for the 1st and 2nd run. We measured the reliability levels of the task by Cronbach’s alpha. The reliability of the entire experiment (1st and 2nd run together) was 0.74, 95% CI [0.62 0.82], for the 1st run only 0.60, 95% CI [0.43 0.74] and for the 2nd run 0.71, 95% CI [0.593 0.814].

### Numerosity perception

Numerosity discrimination thresholds were psychophysically measured with a discrimination task (2AFC). The stimuli were two patches of dots briefly and simultaneously (500 ms) presented on each side of a central fixation point. After the stimuli disappeared, participants touched the side of the screen with more dots. The numerosity of the test stimulus (randomly left or right) was 9, while the probe changed with the method of constant stimuli between 3 and 16 (all numbers within this range presented in random order), ensuring that each stimulus value was presented the same amount of time. Unlike adaptive psychophysical methods, the method of constant stimuli always presents the same stimuli in random order across participants. Thus, children were tested with the whole range of numerosities, avoiding the risk of adaptive algorithms of presenting only a restricted range of stimuli when there are many finger errors. All participants performed three sessions of 42 trials each (126 trials in total for each participant).

Dots were 0.2° diameter, presented at 90% contrast on a grey background of 40 cd/m^2^. Within each array, dots were half white and half black, so that luminance levels did not vary with numerosity. They were constrained to fall within a virtual square of 7° × 7°, centred at 7° eccentricity. The proportion of “test greater” trials was plotted against the numerosity of the probe (on log axis), and fitted with cumulative Gaussian error functions. The difference in numerosity between the 50% and 75% points gives the just notable difference (JND), which was used to estimate discrimination threshold Weber fraction (WF = JND/N_test_) that was then transformed into a measure of normalized sensitivity (1/WF).

WF reliability was measured using a split-half “sample-with-replacement” (non-parametric) bootstrap technique suitable for reliability of measures extracted from psychometric functions^37,38^. For each participant we calculated two separate estimates of sensitivity from a random sample of the data (as large as the data set taken, sampled with replacement from the data set), and then computed the correlation between those two measures. We reiterated the process 10,000 times for all participants, to yield mean and standard error estimates of reliability. Split-half reliability level was 0.66.

## Results

### Non-symbolic geometry, numerosity and formal geometry as a function of age

Non-symbolic geometry was measured with a paradigm adapted from Amalric et al.^26^, illustrated in Fig. 1 and described in methods. We tested each spatial sequence, of varying complexity, twice, on consecutive runs. In the first-run, children observed a cartoon figure jumping over five out of eight spatial locations arranged in a circular array, and were asked to predict the next three locations to complete the geometric sequence. Errors were signalled by a sound, then the program restarted from the beginning of the sequence, including the right position that had been mistaken before. The child had to point to the next locations to complete the whole sequence. In the second run, they had to reproduce entirely the same configuration after being cued with just the first three positions.

**Figure 2:**
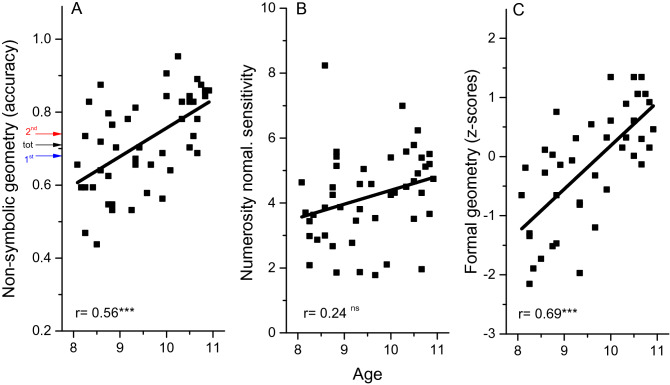
Data from individual participants as a function of age. (**A**) Accuracy in the non-symbolic geometry task. Arrows report average accuracy separately for the aggregate index (black) and for the first (blue) and second runs (red) separately. (**B**) Normalized sensitivity for numerosity. (**C)** Overall performance in the formal geometry battery. Lines report best linear fits. ns > 0.05; ***p < 0.001.

We first evaluated how non-symbolic geometry, numerosity and formal geometry develop with age. Accuracy in non-symbolic geometry (Fig. 2A) varies considerably between individuals (from 0.4 to 0.95), and a significant portion of the variance is explained by age (r = 0.57, p < 0.0001). Average accuracy across runs and spatial configurations was 0.72 ± 0.02 (Fig. 2A, black arrow). As expected, accuracy was higher (t_(48)_ = 3.10, p = 0.003) in the second run (0.74 ± 0.01, Fig. 2 A red arrow) than the first (0.68 ± 0.02, Fig. 2 A blue arrow), probably because the children had become more familiar with the sequence and the task. The accuracy rate in the first and second runs were positively correlated (r = 0.57, p < 0.0001), even when age was controlled for as a covariate (r_p_ = 0.45, p < 0.001). These results suggest that children were well able to perform the task, and that at 10 years of age the mechanisms underlying this intuitive task were still not fully developed. Children capitalized on the experience of the first run to learn and reproduce the spatial configuration in the second run.

Numerosity discrimination was measured by two-alternative forced-choice, with children judging which of two briefly presented dot clouds was more numerous (see methods). Like non-symbolic geometry, numerosity sensitivity (Fig. 2B) also shows large interindividual variability (ranging from around 2 to 8: mean = 4.2). Numerosity sensitivity was generally higher for older children, although the correlation with age did not reach statistical significance (r = 0.25, p = 0.09).

Formal geometry was measured with a battery comprising two factors: *geometrical knowledge*, mainly testing verbal skills (such as “what is a segment?”); and *visuo-spatial abilities*, testing non-symbolic geometric skills, such as solving problems with blocks and shapes (see methods). The z-scores of each were summed to provide an aggregate index (*symbolic geometry index*). Figure 2 C shows that this index depended strongly on age (r = 0.69, p < 0.0001).

### The link between non-symbolic and formal geometry

**Figure 3:**
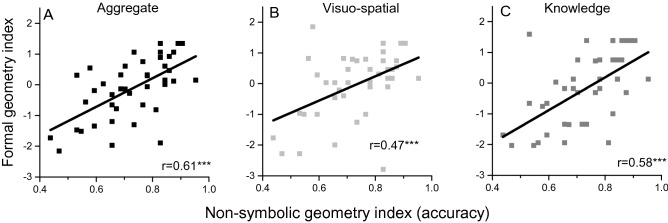
The link between formal and non-symbolic geometry. (**A**) Formal geometry index (z-scores) plotted against z-scores for the non-symbolic, psychophysical geometry test. (**B**) Same as (**A**), but considering only visuo-spatial items in the formal geometry test. (**C**) Same as (**A**), considering only verbal knowledge items. Lines are best linear fits. ***p <= 0.001.

We ran zero-order correlations between performance on non-symbolic and formal geometry scores. Figure 3A shows that the formal geometry index was positively and significantly correlated with non-symbolic geometry (r = 0.61, p < 0.001). Both “visuo-spatial abilities” (Fig. 3B) and “geometrical knowledge” (Fig. 3C) also correlated positively with the overall accuracy in the non-symbolic geometry test (r = 0.47, p = 0.001; r = 0.58, p < 0.001 respectively).

As both non-symbolic and formal geometry covary with age (Fig. 2A,C) we reran the correlations after regressing out age. The partial correlations show that the formal geometry index remained significantly correlated with non-symbolic geometry (r_p_ = 0.37, p = 0.019), as did the “geometrical knowledge” factor (r_p_ = 0.33, p = 0.034). The “visuo-spatial abilities” factor maintained the positive direction but did not reach significance (r_p_ = 0.15, p = 0.11). As described above, non-symbolic geometry was measured in two separate runs, and accuracy improved on the second run. When tested separately, after regressing out age, only accuracy measured in the second run was significantly correlated with formal geometry scores (r_p_ = 0.034, p = 0.82 and r_p_ = 0.50, p = 0.0002 for the first and second runs respectively). The pattern of result remained the same even when splitting the formal geometry scores into “visuo-spatial abilities” and “geometrical knowledge” (first-run Vs geometrical knowledge: r_p_ = 0.004 p = 0.98, Vs visuo-spatial abilities: r_p_ = 0.051 p = 0.75; second-run Vs geometrical knowledge: r = 0.48 p = 0.002, Vs visuo-spatial abilities: r_p_ = 0.325 p = 0.038).

### The link between numerosity perception and formal geometry

**Figure 4:**
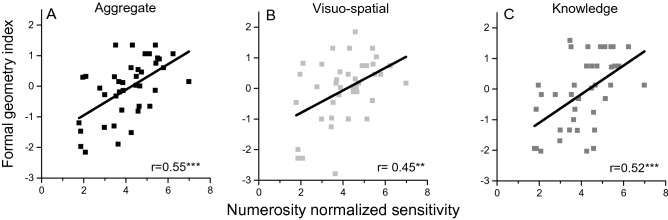
The link between formal geometry and numerosity perception. Z-scores for the formal geometry test plotted as a function of numerosity discrimination sensitivity, considering all the items (**A**), or only visuo-spatial (**B**) or verbal knowledge (**C**). Lines are best linear fit. ***p<=0.001; **<=0.01.

As many previous studies have found positive correlations between numerosity sensitivity and symbolic math abilities^2,12,13^, we investigated whether numerosity perception also predicts formal geometry (Fig. 4).

Zero-order correlations show that numerosity sensitivity was positively correlated with the formal geometry index (r = 0.55, p < 0.001, Fig. 4A). Similarly, numerosity sensitivity was well correlated with both formal geometry factors considered separately (r = 0.45, p = 0.004, Fig. 4B; r = 0.52, p < 0.001, Fig. 4C for visuo-spatial and knowledge respectively). Even after controlling for age the correlations remained statistically significant (formal geometry index: r_p_ = 0.52, p = 0.001; visuo-spatial: r_p_= 0.36, p = 0.014; knowledge: r_p_ = 0.47, p = 0.004).

### Formal geometry and non-symbolic skills: specific and shared variance

As described in the previous paragraphs both numerosity and non-symbolic geometry correlated positively with formal geometrical abilities. We therefore asked whether numerosity and non-symbolic geometry are independent of each other, and whether each of them is an independent predictor of formal geometrical abilities.

We first considered the overall non-symbolic geometry index, combining the performance on the first and second run. The first suggestion of partial independence of numerosity and non-symbolic geometry is that after regressing out age, numerosity sensitivity did not significantly correlate with non-symbolic geometry (r_p_ = 0.23, p = 0.12). We then tested whether non-symbolic geometry and numerosity sensitivity predict formal geometry independently from each other. After controlling for age and for the other factor (either numerosity or non-symbolic geometry), the correlation between formal geometry and numerosity remained robust (r_p_ = 0.48, p = 0.02), while the correlation between formal and non-symbolic geometry lost significance but remained as a trend (r_p_ = 0.30, p = 0.07).

**Figure 5:**
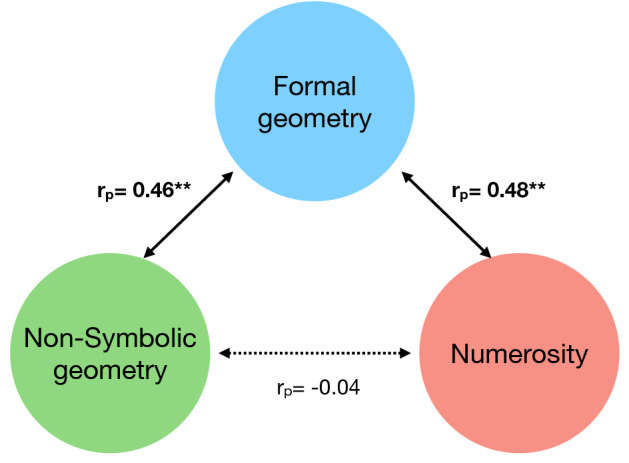
Diagrams of correlations between symbolic and non-symbolic abilities. Values reports partial correlations between the two variables connected by arrows after controlling for age and the third variable. The results refer to the second (most informative) run of the non-symbolic geometry test. **p <= 0.01 p values.

Given that the second run of the non-symbolic geometry test proved to be a far stronger predictor than the first (see above), we repeated the analysis for the second run only (Fig. 5). Again, we found no correlation between numerosity sensitivity and non-symbolic geometry (r_p_ = − 0.04, p = 0.8). After controlling for age and numerosity sensitivity, non-symbolic geometry scores on the second run were strongly correlated with formal geometry scores (r_p_ = 0.46, p = 0.003), and numerosity sensitivity remained significantly correlated with formal geometry (r_p_ = 0.48, p = 0.002) after controlling for age and accuracy measured in second run of non-symbolic geometry.

These results confirm the previous analyses in suggesting that the second run was more informative than the first, and that numerosity perception and non-symbolic geometry may represent partially independent predictors of formal geometry.

In a final analysis we asked whether performance in the two non-symbolic tasks and their ability to predict formal geometry are related to verbal reasoning abilities, measured by the “similarities” sub-test of the WISC-IV battery, in which children are presented with two words and asked how they are related (e.g. how are salt and water related). Interestingly, non-symbolic geometry was positively correlated with the measure of verbal reasoning (r = 0.38, p = 0.006). However, numerosity sensitivity showed no significant correlation (r = 0.05, p = 0.72). This reinforces the idea that the two non-symbolic tasks are relatively independent predictors, and that this specific non-symbolic geometrical task is partially related to verbal reasoning skills. Considering verbal reasoning as a covariate, however, did not annul the correlation between non-symbolic (both first and second runs) and formal geometry (r_p_ = 0.56, p < 0.001), nor that between numerosity and formal geometry (r_p_ = 0.57, p < 0.001). When considering only the second run of the non-symbolic test, the correlation was even higher (r_p_ = 0.65, p < 0.001).

## Discussion

Very few studies have tested whether non-symbolic geometrical abilities predict formal symbolic geometry, an important part of school curricula. In this study we asked whether the spontaneous recognition of geometrical sequential structures (non-symbolic geometry), which could reflect geometrical intuition, is correlated with school-based formal geometrical abilities in primary school children. We also asked whether this relationship is specific, or whether formal geometry is also predicted by numerosity perception, another non-symbolic ability which has been previously shown to be related to arithmetical ability.

The results show that in primary school children, non-symbolic geometry, non-symbolic number (numerosity) and formal geometry performance all tend to improve with age, suggesting that all these components refine relatively late with development and schooling. However, performance in both non-symbolic geometry and numerosity discrimination tasks predicted proficiency in formal geometry over and above these concomitant developmental changes. Interestingly, we found that the second run of the non-symbolic geometry test was much more informative than the first run in predicting formal geometry. It is possible that performance in the first run was too noisy to be a robust predictor, and that during this run children were still becoming familiar with the task, which involved understanding that there is a geometrical rule to follow to correctly complete the sequence. Whatever the reason, the results suggest that the second run is the most informative.

Performance in the non-symbolic geometry and numerosity discrimination tasks predicted proficiency in formal geometry independently of each other: the correlation between scores in non-symbolic geometry (especially on the second run) and formal geometry remained strong when controlling for numerosity sensitivity (in addition to age). Although the correlational nature of the current study does not allow us to infer causality or specificity of the underlying mechanisms, that both non-symbolic geometry and numerosity discrimination independently predict formal geometrical skills motivates future investigation into these links.

That the novel intuitive geometrical reasoning task devised by Amalric et al. predicts formal geometry as assessed by standardized tests opens the path to future studies to compare the efficacy of this test with previous approaches, such as the math game used by Dillon et al^34^ for pre-school screening and training programs (see introduction). Moreover, future studies could test whether training pre-schoolers with the non-symbolic geometrical reasoning task improves geometrical and numerical competences even more, and potentiate the precursor of formal geometry yielding to longer lasting and more generalized effects. These studies could test not only whether training with the non-symbolic task improves formal geometry, but also the reverse. Piazza et al.^39^ found that math education significantly enhanced numerosity discrimination in unschooled Mundurucù participants. It may therefore be interesting to test whether advancing in formal geometrical learning enhances the accuracy of the non-symbolic geometrical test, as much as learning arithmetic sharpens the number sense.

In the current study we also found that performance on the non-symbolic geometrical reasoning task was not the only predictor of formal geometry: numerosity perception also explained a portion of the variance of formal geometric abilities. Even when controlling for age and performance in the non-symbolic geometry test, numerosity thresholds explained a significant portion of the variance of formal geometry performance. This evidence is in partial contrast with the results of Lourenco et al.^32^, who showed that in adults, numerosity discrimination thresholds did not contribute to formal geometry scores when non-symbolic geometry was controlled. Several reasons may account for this difference, the most obvious being the different methods used to measure non-symbolic geometry: average dot-area discrimination thresholds in their case, rather than the ability to extract geometrical regularities from spatial sequences. Numerosity and dot-area discrimination probably share more specific variance than that shared by numerosity discrimination and geometrical spatial sequences perception^40^. However, the different age of participants (17-21 y Vs 7-11 y) may also have led to different conclusions. Lourenco and Bonny^33^ found that for children aged 5-6 years, both numerosity and area explained specific portions of variance in a formal geometry test. These results suggest that the specificity of the predictive link between non-symbolic magnitudes and formal geometry may increase with age (being generalized for children, but specific in adults), similarly to that observed for arithmetic by the same authors. In line with these findings, other studies have shown that the ability to focus on numerosity, as opposed to other non-numerical dimensions, progressively increases with age and arithmetical competence^41–43^.

While we found that numerosity perception predicted symbolic geometry, some caution is needed when considering this effect of numerosity independently from non-numerical features that were not specifically controlled in the current experiment. It could be argued that the ability to discriminate cumulative area of the dots (total ink), rather than numerosity per se, drives the correlation between tasks. This is an ongoing debate, which has been argued extensively in other arenas (for example see ref^44^ and associated commentaries). However, while our current design cannot exclude contamination from non-numeric factors such as surface area, they are unlikely to fully account for the correlations. Firstly, our stimuli were half-white half-black, so cumulative are did not lead to a change in luminance (as in most paradigms). Secondly, many studies have shown that humans are more sensitive to changes in numerosity than in cumulative area since early life: 6-months old infants need up to 4-fold variation to perceive a change in total surface area, while only 2-fold variation is sufficient for them to notice a numerical difference^45^. In adults, numerosity drives decisions in quantity discrimination tasks^46,47^, and biases cumulative and average item area judgments in discrimination tasks^48^ and Stroop-like interference paradigms^41^, suggesting that numerosity is hard to ignore and spontaneously attracts attention^49^. Overall, given that numerosity-linked changes in total surface area have been shown to be less salient than the same changes in number, it appears unlikely that area would be the dimension that drives numerical choices in the current experiment.

The current study showed that non-symbolic geometry was correlated with verbal reasoning skills, but numerosity was not, again pointing to a partial independence of the two non-symbolic tests which separately contribute to formal geometry. What mediates the relation between non-symbolic geometry and a verbal reasoning task remains to be understood. It should also be investigated whether and to what extent the non-symbolic geometry test used here taps cognitive processes such as working memory or visuo-spatial attentional resources and address which of these factors mediate the reported correlation between non-symbolic and formal geometry. It is also interesting that the non-symbolic geometry test exploited was originally designed to test a visuospatial “geometrical language”^26^. The task makes use of recursive rules, a main feature of verbal language. Although speculative, the recursiveness could be a factor linking the performance on the non-symbolic geometrical task and the verbal reasoning scores.

Another possibility is that the link between the non-symbolic geometry and a verbal reasoning is partially mediated by semantics, knowledge of shape names such as the “square” or “rectangle” sequence. A further possibility for the common ground between the two tests lies in reasoning skills. While the reason for the relation between these two tests remains unclear, it is unlikely that they measure the very same construct as controlling for verbal reasoning skills did not annul the correlation between both non-symbolic and formal geometry.

Overall, the current study suggests that non-symbolic geometry and numerosity abilities independently predict formal geometrical skills and this predictive link cannot be explained by age nor by verbal reasoning skills.

Why is formal geometrical knowledge related to the perception of geometrical sequential structures and numerosity perception? One possibility, previously advanced to explain the link between numerosity perception and other functions, including space and time processing^50^, visual motion^51^ and size perception^52^, is “cortical recycling”^53^, the idea that cultural evolution has taken advantage of evolved brain areas to integrate and host new emerging cultural functions such as mathematics. In this way, the correlations found here may arise from shared or highly interconnected brain circuits for formal and non-symbolic geometry, as well as numerosity. In line with this idea, many brain imaging studies have found that both numerosity perception and arithmetical tasks (e.g. mental calculation) in children^54^ and adults^55^ activate similar or nearby^56^ areas within the parietal cortex. Interestingly, a recent imaging study found that the parietal areas activated by arithmetical tasks were also recruited by the non-symbolic geometry test used here^57^.

Overall, the relation between non-symbolic and symbolic systems still clearly needs further investigation. The current study contributes to the existing literature by suggesting that non-symbolic numerosity together with non-symbolic geometry, as measured by the novel non-symbolic geometrical reasoning task devised by Amalric et al, constitute the building blocks for the development of formal geometrical skills in primary school children.

## Data Availability

Data used in this study have been deposited into the Zenodo Repository: https://zenodo.org/record/4544388#.YC0wuRNKiuU, https://doi.org/10.5281/zenodo.4544388.
